# Macular Pigment Changes and Visual Recovery Following Successful Full-Thickness Macular Hole Closure Using the Inverted Flap Technique

**DOI:** 10.3390/jcm14010290

**Published:** 2025-01-06

**Authors:** Michele Rinaldi, Nicola Galantuomo, Maria Laura Passaro, Gilda Cennamo, Flavia Chiosi, Ciro Costagliola

**Affiliations:** 1 Department of Neurosciences, Reproductive Sciences and Dentistry, University of Naples “Federico II”, 80131 Napoli, Italy; 2 Department of Medicine and Health Sciences “V. Tiberio”, University of Molise, 86100 Campobasso, Italy; 3 Department of Ophthalmology, Azienda Ospedaliera dei Colli, Monaldi Hospital, 80131 Napoli, Italy

**Keywords:** macular pigment optical density, full-thickness macular hole, visual recovery

## Abstract

**Objectives**: This study aimed to assess the role of macular pigment optical density (MPOD) in patients with a full-thickness macular hole (FTMH) compared to healthy controls, evaluating postoperative changes in MPOD and exploring potential correlations with visual outcomes. **Methods**: This prospective, cross-sectional, comparative study included 16 eyes from FTMH patients who achieved anatomical hole closure following pars plana vitrectomy with the inverted ILM flap technique. Each eye underwent a comprehensive ophthalmologic examination, including BCVA and intraocular pressure measurements, anterior segment evaluation, fundus examination, and macular assessment with Enhanced Depth Imaging Optical Coherence Tomography (EDI-OCT, Spectralis, Heidelberg Engineering Inc., Heidelberg, Germany). Macular pigment optical density (MPOD) was measured using one-wavelength reflectometry (Visucam 200, Zeiss Meditec, Jena, Germany). These evaluations were conducted preoperatively and at 1, 3, and 6 months postoperatively to assess changes over time and correlate MPOD with visual outcomes. **Results**: Significant baseline differences were observed between FTMH patients and controls for BCVA, mean MPOD, maximum MPOD, and MPOD volume (*p* < 0.05). Postoperative BCVA improved significantly (*p* = 0.0011), with a notable increase in MPOD volume at 6 months (*p* = 0.01). A positive correlation was found between BCVA improvement and MPOD volume increase (r = 0.739; *p* = 0.002). **Conclusions**: In conclusion, MPOD measurement may serve as a valuable addition to the follow-up of FTMH surgery, providing insights into photoreceptor function and macular metabolic activity, potentially correlating with visual recovery. Further longitudinal studies are needed to clarify its relationship with clinical variables, such as metamorphopsia and OCT microstructural findings.

## 1. Introduction

Full-thickness macular hole (FTMH) is a severe retinal disorder characterized by a defect in the foveal region, which leads to substantial central vision loss and metamorphopsia [1]. Affecting approximately 0.09% of the population [2], FTMH predominantly arises in individuals in their sixth decade of life [3], with a higher incidence reported among women [4]. If left untreated, FTMH can result in irreversible visual impairment [5]. Advancements in surgical techniques, including pars plana vitrectomy with internal limiting membrane (ILM) peeling and the inverted flap technique, have greatly improved closure rates for FTMH, thereby enhancing visual outcomes for many patients [6]. Despite these advancements, some individuals experience incomplete visual recovery and persistent metamorphopsia due to subtle structural abnormalities that may remain postoperatively [7].

In recent years, optical coherence tomography (OCT) and fundus autofluorescence (FAF) imaging have become valuable tools in assessing this structural recovery. In addition to structural evaluation, there is growing interest in functional markers, such as macular pigment optical density (MPOD), which may provide further insights into photoreceptor health and metabolic activity within the macula.

The aim of this study is to evaluate differences in macular pigment optical density (MPOD) between patients with full-thickness macular hole (FTMH) and healthy controls, to assess postoperative changes in MPOD, and to correlate these changes with visual prognosis. The significance of this approach lies in its potential to enhance our understanding of both structural and functional recovery in FTMH patients, providing valuable insights into the role of MPOD as a prognostic marker that complements traditional imaging techniques.

## 2. Materials and Methods

### 2.1. Study Design and Setting

This prospective, cross-sectional, comparative study was conducted at the Eye Clinic of the University of Naples “Federico II” between July 2023 and April 2024. This study was retrospectively registered on ClinicalTrials.gov (NCT06664515) and adhered to the principles outlined in the Declaration of Helsinki. Written informed consent was obtained from all participants before enrollment, with strict adherence to confidentiality and privacy standards.

### 2.2. Participants

**Figure 1:**
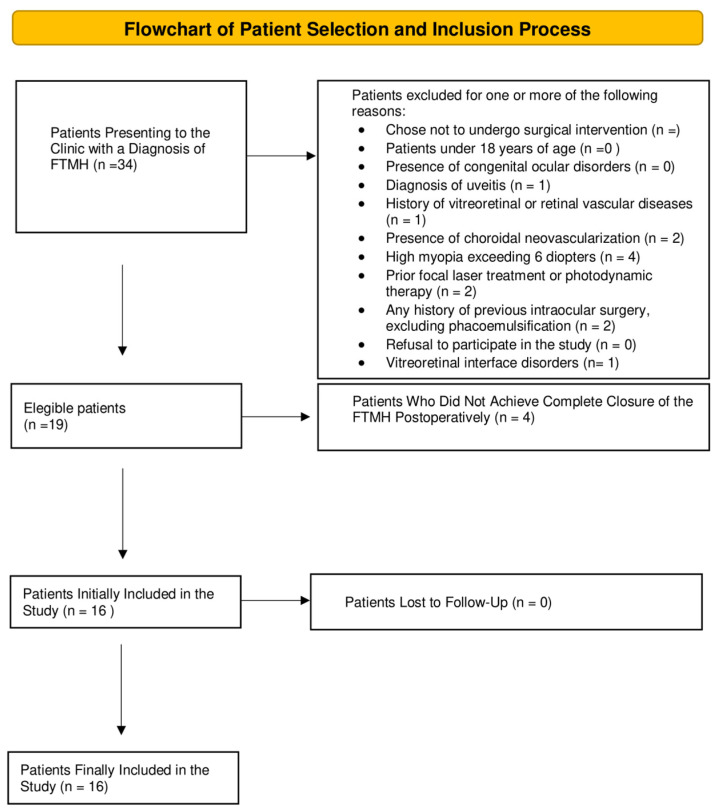
Flowchart illustrating the process of patient selection and inclusion in the study.

Patients aged 18 years or older who were referred to the vitreoretinal service with a diagnosis of full-thickness macular hole (FTMH) and underwent surgery using the inverted ILM flap technique, achieving complete post-operative hole closure, were included in this study (Figure 1). Exclusion criteria encompassed congenital ocular disorders, uveitis, a history of vitreoretinal or retinal vascular diseases, choroidal neovascularization, high myopia (greater than 6 diopters), prior focal laser treatment or photodynamic therapy (PDT), and any previous intraocular surgery.

### 2.3. Surgical Technique

All surgical procedures were performed by an experienced vitreoretinal surgeon (M.R.) following the inverted ILM flap technique. After removing the epiretinal membrane, the ILM was grasped with forceps and peeled in a circular manner, covering an area approximately 2 disk diameters around the macular hole. The ILM edges were then trimmed with a vitreoretinal cutter, and the remaining flap was inverted to cover the macular hole, following the technique described by Michalewska et al. [6]. An air tamponade was applied to the globe, and patients were instructed to maintain a face-down position for 6 days for 7 h each day.

### 2.4. Retinal Imaging and Data Measurement

Each eye underwent a complete ophthalmologic examination, including BCVA measurement, intraocular pressure measurement, anterior segment evaluation, fundus examination, and macular assessment using Enhanced Depth Imaging Optical Coherence Tomography (EDI-OCT, Spectralis, Heidelberg Engineering Inc.). Moreover, macular pigment optical density (MPOD) was assessed using the one-wavelength reflectometry method (Visucam 200 Zeiss Meditec, Jena, Germany). These assessments were performed preoperatively and subsequently at 1 month, 3 months, and 6 months postoperatively to systematically evaluate changes over time.

### 2.5. Macular Pigment Assessment and Analysis

The assessment of MPOD was conducted using the one-wavelength fundus reflectance method [8,9], with the Visucam 200 (Zeiss Meditec, Jena, Germany). This specialized fundus camera utilizes narrow-band blue light reflectance to estimate macular pigment (MP) levels, as areas with MP absorb blue light more intensively than surrounding retinal regions. In the blue reflectance images, areas of greater absorption appear darker, indicating the higher optical density of the MP.

Participants were instructed to position their foreheads against the top bar and their chins on the chin rest for head stabilization, with chin-head straps ensuring alignment. An internal fixation target, centrally located, aided image alignment. Pupil dilation was achieved using one drop of 1% tropicamide, and 45° color fundus photographs were taken 30 min after dilation. The retina was illuminated with blue light, capturing the MPOD image solely through the blue channel to minimize interference from autofluorescence in the green spectrum.

The MPOD measurement was performed over a 30° field within the fundus photograph, with automatic flash settings and flash intensity adjusted to 12. Autofocus was enabled. MPOD values were analyzed in the region spanning 4–7° of eccentricity around the fovea, encompassing the primary distribution of xanthophyll pigments. This was differentiated from the background reflection beyond 7° of eccentricity, where MP is absent. Optical density and MP distribution were calculated with specialized software, recording the following metrics for each image: (a) mean MPOD and reproducibility; (b) maximum MPOD value; (c) MPOD area—the area within which the MP was detected and defined on the background; and (d) MPOD volume—the cumulative optical density across the MPOD area. Mean MPOD reflects the average density of xanthophylls across the surface area, while maximum MPOD represents the highest density, typically found at the foveal center. MPOD is reported in density units (du).

### 2.6. Variables and Outcomes

Baseline data from patients were compared with those of healthy age-matched controls. The controls were recruited among healthy individuals undergoing routine check-ups and who met the same exclusion criteria as patients with FTMH, including the absence of congenital ocular disorders, uveitis, a history of vitreoretinal or retinal vascular diseases, choroidal neovascularization, high myopia (greater than 6 diopters), prior focal laser treatment or PDT, and any previous intraocular surgery. Subsequently, differences in best-corrected visual acuity (BCVA), mean MPOD, maximum MPOD value, MPOD area, and MPOD volume were evaluated between the postoperative period and at the 6-month follow-up. Finally, associations between MPOD values and both anatomical and functional changes were thoroughly analyzed.

### 2.7. Statistical Analysis

Statistical analysis was conducted using Stata 11 software (StataCorp, 2009, Stata Statistical Software: Release 11, College Station, TX, USA). An unpaired t-test was used to assess differences between study groups. Pearson correlation coefficients (r) were calculated to determine the strength and direction of associations between mean MPOD and best-corrected visual acuity (BCVA). Both linear and nonlinear (second-order) regression models, with analysis of variance (ANOVA), were used to further examine these relationships. A *p*-value of less than 0.05 was considered statistically significant.

## 3. Results

**Figure 2:**
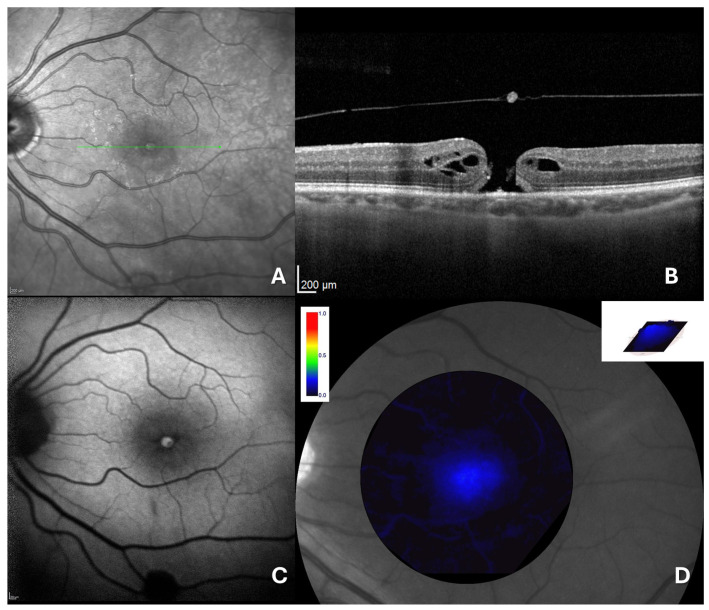
(**A**) En face OCT image demonstrates FTMH in the foveal area; (**B**) OCT imaging of the macula reveals FTMH with marked disruption of all retinal layers, without vitreomacular traction; (**C**) autofluorescence imaging reveals iperautofluorescence in macular region; (**D**) MPOD appears significantly reduced.

A total of 16 eyes from 16 patients were included in this study. The mean age of the participants was 61.5 ± 8.7 years. The mean preoperative BCVA was 1.5 ± 0.5 logMAR. All patients had intraocular pressure (IOP) ≤18 mmHg. Anterior segment examination revealed clear corneas and normally reactive pupils. Seven patients (43.8%) were pseudophakic, while the remaining nine (56.2%) were phakic. In all patients, fundus examination demonstrated a large FTMH, confirmed through multimodal imaging including en face OCT (Figure 2A), spectral-domain OCT (Figure 2B), and autofluorescence (AF) (Figure 2C).

The mean FTMH size was 985 µm ± 150 µm. MPOD analysis yielded the following values: mean MPOD of 0.1 ± 0.02 d.u., maximum MPOD of 0.4 ± 0.08 d.u., MPOD area of 63,597.1 ± 16,739.2 pixels, and MPOD volume of 10,812.6 ± 3329.7 d.u. × pixels.

All included patients underwent surgery using the inverted ILM flap technique, with additional phacoemulsification and intraocular lens (IOL) implantation for phakic patients, achieving complete closure of the macular hole in all cases.

At baseline, significant differences were observed between the FTMH group and the age-matched control group in terms of BCVA (*p* < 0.001), mean MPOD (*p* < 0.001), maximum MPOD (*p* = 0.002), and MPOD volume (*p* = 0.0006) (Table 1).

**Figure 3:**
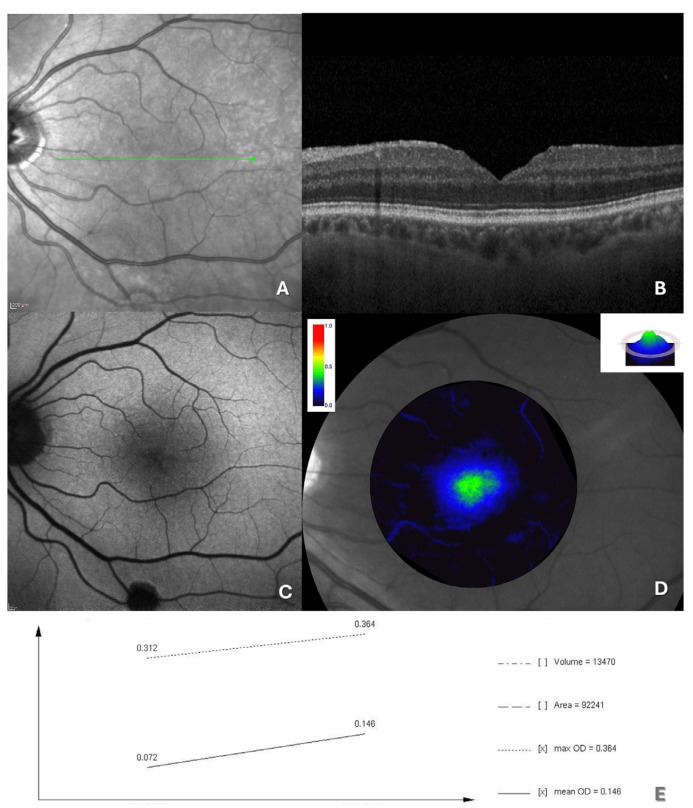
(**A**) En face OCT reveals a well-closed macular hole (the green arrow indicates the location and orientation of the cross-sectional scan shown in panel **B**); (**B**) OCT imaging demonstrates a regular retinal contour, and recovery of central foveal depression, with the successful closure of the macular hole; (**C**) autofluorescence imaging reveals hypoautofluorescence in the macular region; (**D**) post-operative MPOD measurements show improved values; (**E**) the line graph illustrates the temporal variations in the metrics of interest for MPOD, including maximum optical density (max OD), mean optical density (mean OD), MPOD area, and MPOD volume. The arrow in (**A**) is were is conducted the OCT scan in (**B**).

At the 6-month follow-up after surgery, all 16 patients maintained macular hole closure (Figure 3).

BCVA showed a marked improvement, increasing from 1.5 ± 0.5 logMAR to 0.7 ± 0.4 logMAR (*p* = 0.0011). Notably, while most MPOD parameters did not show statistically significant changes postoperatively, MPOD volume exhibited a significant increase, rising from 10,812.6 ± 3329.7 d.u. × pixels to 14,569.1 ± 4764.6 d.u. × pixels (*p* = 0.01). All data regarding MP measurements are provided in Table 2.

Interestingly, an analysis of correlations between postoperative changes in BCVA and MPOD volume demonstrated a strong positive correlation (r = 0.739; *p* = 0.002), suggesting a potential link between functional recovery and MP levels following FTMH surgery.

## 4. Discussion

Macular pigments (MP), primarily lutein and zeaxanthin, are vital antioxidants localized within the macular region. These pigments play a critical role in filtering blue light and neutralizing free radicals, thereby safeguarding retinal tissue from oxidative damage that may otherwise contribute to the development of various retinal pathologies [8,10]. These carotenoids play a vital role in neutralizing reactive oxygen species (ROS), which are by-products of oxidative stress that can damage the photoreceptor outer segments and the retinal pigment epithelium. By mitigating this oxidative damage, they help preserve the structural and functional integrity of retinal tissues. Additionally, lutein and zeaxanthin absorb high-energy blue light, reducing its potential to cause photochemical damage to the retina and thereby maintaining visual acuity and retinal health. Moreover, these carotenoids have also been suggested to offer protection to reduce lipofuscin accumulation and enhance lysosomal stability and viability [11].

Anatomically, MP is primarily concentrated in the Henle fiber layer, the foveal region of the outer plexiform layer (OPL), where cone axons are densely packed. Additional pigment is also present in the inner plexiform layer (IPL) of the foveola, within a specialized inverted cone-shaped zone of Müller cells, referred to as the “Müller cell cone” by Gass [8,12,13,14]. Obtained through dietary intake, lutein and zeaxanthin are believed to play a significant role in preventing or delaying the onset of age-related macular degeneration, supporting the potential for dietary interventions as a preventative strategy [8].

Obana et al. proposed that MP is composed of two parts: MP in the foveolar Muller cell cone and MP in the Henle fibers layer at the fovea, with distribution patterns that vary among individuals: central peak, ring-like, plateau, and central dip distribution [14]. Notably, a central dip distribution and lower MP levels at the foveal center may indicate Müller cell damage and serve as an early predictor of macular hole development [14].

Post-surgical observations further reinforce the value of MP in evaluating recovery. FTMH surgery, which restores the anatomic integrity of the fovea, often leads to MP re-accumulation and visual improvement [15,16].

Several studies have demonstrated that visual recovery is closely linked to anatomical restoration, particularly the continuity of the inner segment/outer segment (IS/OS) line on OCT, which is strongly correlated with better visual outcomes [17,18]. FAF has similarly shown that decreased autofluorescent signals in closed maculae after surgery are associated with better visual prognosis [19].

These findings indicate that MPOD may serve as a valuable complementary marker alongside OCT and FAF, offering additional insights into photoreceptor function and macular metabolic activity during visual recovery. In contrast to other techniques such as OCT, which primarily address structural aspects, MPOD provides a distinctive functional perspective on photoreceptor recovery and macular health.

Several methods exist for investigating and measuring macular pigment, including Raman spectroscopy, heterochromatic flicker photometry, fundus autofluorescence imaging, and fundus reflectometry. In particular, fundus autofluorescence assesses MP levels based on the attenuation effect of MP on fluorescent light emitted by lipofuscin in the retinal pigment epithelium (RPE) [20].

In 2016, Bottoni et al., using Raman spectroscopy, demonstrated that, in a cohort of 18 consecutive patients who had undergone macular hole surgery, MP levels were reduced compared to age-matched healthy controls, with re-accumulation of macular pigment observed following successful macular hole closure [21].

Romano et al. emphasized the accessibility and repeatability of MP measurements with the one-wavelength reflectance method of Visucam 200 Zeiss fundus camera, which demonstrated a marked reduction in MP in cases of vitreoretinal interface disorders, including FTMHs [20]. The authors highlighted that this reduction in pigment in FTMH was closely associated with the pathophysiology of the condition, particularly the opening at the fovea and the subsequent centrifugal displacement of the MP. A statistically significant increase in MPOD volume post-surgery in patients with FTMH was also observed. They hypothesized that closure of the macular hole surface facilitates the migration of cone photoreceptors and their axons, leading to the re-accumulation of macular pigment in the foveal center [20]. This reappearance and increase in MP following surgery indicate a positive sign of physiological recovery, suggesting not only structural repair, but also a restoration of photoreceptor functionality and macular integrity—factors that are expected to be associated with improvements in postoperative visual acuity.

Jordan et al. further supported these findings, demonstrating that patients with idiopathic macular holes showed significant increases in maximum MP optical density and MP volume following pars plana vitrectomy with dye-assisted peeling of the internal limiting membrane. These increases were especially pronounced in patients with stage 4 FTMH [22].

While advanced techniques, such as pars plana vitrectomy with ILM peeling and the inverted flap technique, have improved success rates for FTMH repair, some patients experience limited visual recovery and persistent metamorphopsia due to subtle structural abnormalities that remain postoperatively [7].

However, the relationship between postoperative MP increase and visual acuity remains an area of active research. The observed association between visual recovery and postoperative MP volume increases, especially when accompanied with IS/OS continuity, strengthens the case for MPOD as a practical and supportive tool for tracking visual prognosis in FTMH patients [23,24].

Based on these findings, we previously reported a single case of an FTMH patient who underwent successful surgery, where we observed an increase in MPOD following the procedure. This case suggested MPOD’s potential role as a valuable adjunctive biomarker associated with favorable visual prognosis after such interventions. Consequently, we expanded our research to a larger cohort of patients to seek more robust evidence supporting this association [16]. Our findings align with this view, as we observed that MPOD volume increases were consistently correlated with improvements in BCVA in a homogeneous group of stage 4 FTMH patients who underwent 25G pars plana vitrectomy with ILM peeling. This suggests that MPOD, with its simplicity and repeatability, could be a meaningful measurement to integrate into follow-up protocols for macular hole surgery, offering a prognostic visual assessment that complements traditional methods.

In our study, only Caucasian patients were analyzed. Interestingly, several studies demonstrates that ethnicity plays a role in MPOD values and in MP distribution [25], reporting racial differences, with Caucasians showing significantly lower MPOD levels compared to African Americans and South Asians [26]. Furthermore, recent studies have shown that the ability to respond to lutein, reflected in blood and tissue concentrations, is at least partially genetically determined and is largely influenced by a combination of SNPs in 15 genes involved in lutein and chylomicron metabolism [27]. Despite the potential preoperative variability in MPOD due to geographical, ethnic, or genetic factors, our focus on the variation in pigment density ensures that interindividual variability does not affect the interpretation of our results. Nonetheless, future studies involving diverse populations would be valuable to further explore these differences and their potential implications.

In conclusion, MPOD measurement holds promising potential as a valuable addition to the follow-up protocol for patients undergoing FTMH surgery, offering additional insights into visual recovery. MPOD measurement provides a comprehensive assessment that extends beyond evaluating structural repair, offering valuable insights into the recovery of photoreceptor functionality and the re-establishment of macular integrity. As this method is simple, repeatable, and complementary to established imaging techniques, further longitudinal studies are essential to explore in detail the correlation between MPOD changes and specific clinical variables, such as the degree of metamorphopsia and microstructural OCT findings. Confirming this correlation would open new perspectives on the diagnostic and prognostic approaches to macular holes and other macular pathologies.

## Figures and Tables

**Table 1:** Characteristics of the study population. FTMH = full-thickness macular hole; BCVA = best-corrected visual acuity; MPOD = macular pigment optical density; d.u. = density units. Numbers in brackets are standard deviations. *p* values in bold indicate statistical significance.

	Control (16 Eyes)	FTMH (16 Eyes)	*p* Value
Age	62.6 (7.4)	61.5 (8.7)	0.97
BCVA (logMAR)	0.05 (0.1)	1.4 (0.5)	<0.001
MPOD mean (d.u.)	0.15 (0.02)	0.1 (0.02)	<0.0001
MPOD max (d.u.)	0.47 (0.06)	0.4 (0.08)	0.002
MPOD area (pixel)	80,628.8 (8282.6)	63,597.1 (16,739.2)	0.03
MPOD volume (d.u. × pixel)	16,338.3 (2822)	10,812.6 (3329.7)	<0.001

**Table 2:** Macular pigment optical density changes after surgery. FTMH = full-thickness macular hole; BCVA = best-corrected visual acuity; MPOD = macular pigment optical density; d.u. = density units. Numbers in brackets are standard deviations. *p* values in bold indicate statistical significance.

	FTMH		
	Preoperative	Postoperative	*p*-Value
BCVA (logMAR)	1.5 (0.5)	0.7 (0.4)	0.0011
MPOD mean (d.u.)	0.1(0.02)	0.1 (0.01)	>0.9
MPOD max (d.u.)	0.4 (0.08)	0.4 (0.03)	>0.9
MPOD area (pixel)	63,597.1 (16,739.2)	79,982.5 (25,703.0)	0.2
MPOD volume (d.u. × pixel)	10,812.6 (3329.7)	14,569.1 (4764.6)	0.01

## Data Availability

The original contributions presented in this study are included in the article; further inquiries can be directed to the corresponding authors.
